# Golden Ratio Genetic Algorithm Based Approach for Modelling and Analysis of the Capacity Expansion of Urban Road Traffic Network

**DOI:** 10.1155/2015/512715

**Published:** 2015-01-31

**Authors:** Lun Zhang, Meng Zhang, Wenchen Yang, Decun Dong

**Affiliations:** Key Laboratory of Road and Traffic Engineering, Ministry of Education, Tongji University, 4800 Cao'an Road, Shanghai 201804, China

## Abstract

This paper presents the modelling and analysis of the capacity expansion of urban road traffic network (ICURTN). Thebilevel programming model is first employed to model the ICURTN, in which the utility of the entire network is maximized with the optimal utility of travelers' route choice. Then, an improved hybrid genetic algorithm integrated with golden ratio (HGAGR) is developed to enhance the local search of simple genetic algorithms, and the proposed capacity expansion model is solved by the combination of the HGAGR and the Frank-Wolfe algorithm. Taking the traditional one-way network and bidirectional network as the study case, three numerical calculations are conducted to validate the presented model and algorithm, and the primary influencing factors on extended capacity model are analyzed. The calculation results indicate that capacity expansion of road network is an effective measure to enlarge the capacity of urban road network, especially on the condition of limited construction budget; the average computation time of the HGAGR is 122 seconds, which meets the real-time demand in the evaluation of the road network capacity.

## 1. Introduction

The growing demand of urban traffic can never be solved by just increasing road facility. Factors like city economics, road structure, and land use will determine the travel mode, travel path, and average travel distance. In addition, in most cities, the distribution of land used has been decided, and the land values promote high-strength development. Moreover, the newly constructed roads will reduce the travel time but also attract traffic flows from other roads, as well as create the new traffic demand. The road network may return to the original congestion level after a period of time [1]. All these lead to the difficulty of extension and transformation of the existing transportation network [2]. Therefore, three problems, (1) how to analyze capacity of road network, (2) how to evaluate traffic supply conditions and road construction level, and (3) how to decide the scale of new construction and reconstruction of existing network capacity, are key for sustainable development of road infrastructures.

In the aspect of the capacity of road network, experts around the world have proposed different methods to define and calculate the capacity of network, such as graph theory method [3], space-time consume method [4], mathematical programming method (including linear programming method and bilevel programming method) [5], and traffic simulation method [6, 7]. As the capacity of road network is not only a physical network problem, but also a dynamic problem which considers people, as well as delay and costs, both of which change with traffic flows. The travelers' routing choice behavior and traffic state in the network have significant influence on the capacity of road network [8]. In these methods, many scholars have found the great importance of OD pattern on calculating the capacity of road network. Therefore, applying the bilevel mathematical modelling method on describing the traffic capacity of network and developing efficient solution algorithm becomes research focus. Asakura and Kashiwadani proposed the first model about road network capacity balance and the traffic simulation distribution method [9]. Yang et al. combined traffic distribution and assignment model, and they considered the routing choice and destination of travelers, the physical traffic capacity, and environment of each road as the constraint condition of the capacity of road network. An advanced bilevel traffic assignment method was proposed, which considered not only the physical capacity of road network, but also the balance among traffic individuals [10]. The study offers a new method to calculate the road network capacity model.

Despite the promising progress from network topology and network capacity, effective models development and efficient strategies for urban road network capacity remain to be challenged, especially regarding the following issues: (1) network capacity modeling: various network capacities are defined for different design purposes, and these studies analyzed examples of network design problem so as to optimize the road network capacity; (2) model solution: many algorithms have been proposed to calculate the balance model, such as incremental assignment method and Frank-Wolf algorithm, and so forth, but the applications of these algorithms are limited because of too many variables and constraints. So the intelligent optimization algorithms with low complexity are needed to meet the application requirements of large-scale network design.

This paper uses bilevel programming to model the capacity expansion of road network, and an improved hybrid genetic algorithm integrated with golden ratio is developed to solve the upper-level model of capacity expansion, while the lower-level user optimized equilibrium model is solved by classic Frank-Wolfe algorithm. The remainders of this paper are as follows.  Section 2 defines the research scope and assumptions.  Section 3 models capacity expansion of road network.  Section 4 illustrates the solution algorithm combining the golden ratio based genetic algorithm and Frank-Wolfe algorithm.  Section 5 evaluates the proposed model and algorithm with numerical analysis of a classic network.  Section 6 concludes the work.

## 2. Research Scope and Assumptions

### 2.1. Research Scope

The generalized concept of capacity expansion of road network is as follows: through improving influencing factors under the condition of certain economic constraints and geographical environment, the maximum number of traffic volume passing the road section in unit time are increased. These improved measures include, but are not limited to, the road conditions of network, the matching degree of OD distribution and road network structure, the road network layout and hierarchy, the service level of road network, and the route choice behavior of traffic individual. This general concept fully considers the various influence factors of network capacity expansion, but it is not realistic to improve the conditions of each influence factor. For example, changing the road network layout and hierarchy cannot be achieved in short-term management and operation, and optimizing the route choice behavior of traffic individual is very difficult to practice due to subjective factors.

Based on discussions above, this paper mainly focuses on improving road conditions of network as a way of expanding capacity of road network. The narrow definition of expanding capacity of road network is that through improving the road conditions of network under the condition of certain economic constraints and geographical environment, the maximum number of traffic volume passing the road section in unit time is increased, and the dimension is pcu/h.

### 2.2. Assumptions

According to the narrow concept of expanding capacity of road network, what road sections and intersections are taken as the expansion objects must be determined firstly. Following this, the expansion objects of capacity of road network are divided into three categories: (1) take a certain road section or an intersection of the network as the expansion objects; (2) take all road sections and intersections of the network which flow is greater than or equal to the capacity as the expansion objects; (3) take the critical road sections and intersections of the network as the expansion objects. The intersection capacity expansion is mostly improved by traffic organization optimization and traffic designs under the given traffic demand. To do this, the capacity expansion of critical road sections must be first integrated cooperatively. As a foundation of the expansion of intersection capacity and to simplify the combination optimization problem, this paper just selects the set of critical road sections as the expansion objects.

To extend the capacity of road network, urban road network design problem (NDP) is usually divided into following three types [11]: continuous network design problem (CNDP), discrete network design problem (DNDP), and mixed network design problem (MNDP). The CNDP aims to improve the capacity performance of existing road sections by enlarging new lanes. The DNDP aims to extend existing road network by constructing new roads. The MNDP is the combination measures of the CNDP and the DNDP. The corresponding assumptions in this paper are as follows.

1. According to the research scope of the capacity expansion of critical road sections, this paper discusses the type of the CNDP.
2. As the OD demand from those built facilities of a city is relatively stable in a short time, the OD traffic demand in the network is static.

## 3. Modelling Capacity Expansion of Road Network

The routing choices of travelers are mainly determined by the lowest travel cost. But traffic managers expect to optimize the network performance, such as reducing traffic congestion and maximizing the throughput. Therefore not only travel cost of travelers, but also usage of network capacity should be taken into account in routing choice. Following this, here, the bilevel programming model is used to model the travel objectives of both travelers and traffic managers.

The expanded capacity of road network, defined in upper-level model, is to realize global optimization, and the routing choice behavior, denoting by *v*(**u**), is defined in lower-level model. To facilitate the model presentation, the notations used here and after are summarized in Notations section. 

*(1) The Upper-Level Model*. Travel time is a comprehensive index used to evaluate the congested and comfortable level of user's trip. This paper uses the total travel time as the global optimization objective. It is expected that total travel time is reduced with the network capacity expansion. Following this, the upper model of road network capacity is as the following formulas:

(1)  min⁡u Z=∑a∈Atava,ua·va

(2)  s.t.min⁡u∑a∈A−gaua≤B

(3)  min⁡u 0≤ua≤uamax⁡, ∀a∈A−.

Formula (1) minimizes the total travel time in the network. Formula (2) makes sure that the increase of capacity will never exceed the feasible budget. Formula (3) shows the upper limit and lower limit of increasing capacity of road sections. To eliminate the constraints of budget, Lagrange transform is used to simplify the upper model, as shown in formulas (4) and (5). Consider the following:

(4)  min⁡u Z=∑a∈Atava,ua·va+γ∑a∈Agaua,

(5)  s.t.min⁡u0≤ua≤uamax⁡, ∀a∈A−.

In formula (4), *γ* is the Lagrange multiplier.

*(2) The Lower-Level Model*. In a fixed demand traffic assignment problem, with the given expanded capacity of links and fixed demand between OD pairs, the lower model is a standard user equilibrium model, which describes the user's routing choice behavior, as in the following formulas:

(6)  min⁡v ∑a∈A∫0vata(x,ua)·dx

(7)  s.t.min⁡v∑r∈Rwfrw=qw, ∀w∈W

(8)  min⁡v va=∑w∈W∑r∈Rwfrwδarw, ∀a∈A

(9)  min⁡v frw≥0, ∀r∈Rw,  w∈W.

Formula (6) describes the assignment problem of Wardrop user equilibrium (UE). Formula (7) meets the need of the conservation of traffic flow. Formula (8) shows the relationship between section and route traffic. Formula (9) describes the nonnegativity of route traffic. The optimal solution **f** ^*^ = (…, *f*_*r*_^*w*^,…)^*T*^ meets the user equilibrium condition as in formula (10). When the travel time of route *r* is more than or equal to the minimum travel time, the route traffic is zero. When the travel time of route *r* is equal to the minimum travel time, the route traffic is more than zero; that is to say, this route is occupied:

(10) crwf∗−πwf∗=0,if  frw>0,≥0,if  frw=0,iiiiiiiiiiiiiiiiiiiiiiii∀r∈Rw, w∈W.

In formula (10), *c*_*r*_^*w*^(**f** ^*^) = ∑_*a*_ *t*_*a*_(*v*_*a*_)*δ*_*ar*_^*w*^ means the travel time of route *r* in the OD pair numbered *w*. *π*_*w*_(**f** ^*^) = min⁡{*c*_*r*_^*w*^(**f** ^*^), ∀*r* ∈ *R*_*w*_} means the minimum travel time of routes between the OD pair numbered *w*.

## 4. Solution Algorithm

Variables of capacity expansion in upper-level model are necessary to solve lower-level model. Thus, upper-level mathematical model is not a linear optimization model, which is hard to solve by traditional integral equation method as least square method. Genetic algorithm (GA) does not depend on gradient information and experiential knowledge and is able to find global optimum. And, hence, Yin used genetic algorithm to solve network design problem, and introduced bionic mechanisms such as simulated annealing, ants feeding, and particle swam preying to improve the local search ability of simple genetic algorithms [12]. However, the improved genetic algorithm has disadvantages as complicated structure or large calculation, which may cause inefficiency and poor portability. Considering nonlinearity and nonconvexity of bilevel expansion models, this paper introduces the golden ratio to integrate with an improved genetic algorithm to solve upper-level model, and the classic Frank-Wolfe algorithm is used to solve lower-level model.

### 4.1. Frank-Wolfe Algorithm

Use Frank-Wolfe algorithm to calculate the lower-level model under fixed OD travel demand. Main steps are as follows [13].

**Step 1 (initialization).**

Set the iteration number *n* = 1 and find a feasible traffic mode {*v*_*a*_^(1)^}.

**Step 2 (update the travel time).**

Calculate *t*_*a*_^(*n*)^ = *t*_*a*_(*v*_*a*_^(*n*)^), ∀*a* ∈ *A*.

**Step 3 (find direction).**

According to *t*_*a*_^(*n*)^, use all-or-none (AON) algorithm to get the auxiliary flow collection *y*_*a*_^(*n*)^.

**Step 4 (displacement distance).**

As in formula (11), find a better *α* ^(*n*)^along the direction of the objective function minimized:

(11) $$\begin{array}{c} \underset{\alpha^{\left(n\right)}}{\min}\sum_{a\in A}\overset{v_{a}^{\left(n\right)}+\alpha^{\left(n\right)}\left(y_{a}^{\left(n\right)}-v_{a}^{\left(n\right)}\right)}{\underset{0}{\int }}t_{a}\left(x,u_{a}\right)dx. \end{array}$$

**Step 5 .**

Update road traffic network flow, which is to calculate new traffic volume in links as in the following formula:

(12) $$\begin{array}{c} v_{a}^{\left(n+1\right)}=v_{a}^{\left(n\right)}+\alpha^{\left(n\right)}\left(y_{a}^{\left(n\right)}-v_{a}^{\left(n\right)}\right),\;\forall a\in A. \end{array}$$

**Step 6 (judge the end condition).**

If the algorithm reaches the specific judging criteria (such as maximum iterations), end the Frank-Wolfe algorithm. Otherwise *n* = *n* + 1 and returns back to Step 1.

### 4.2. Golden Ratio-Based Heuristic Genetic Algorithm

A golden ratio-based heuristic genetic algorithm (GRGA) has been proposed to yield approximate solutions for expanded capacities of urban road network [14]. The proposed heuristic is able to find the closest solution to the best solution by introducing golden ratio (GR) to enhance the local optimal capability of an improved real-coded genetic algorithm [15].

*(1) Golden Ratio Based Local Search*. When genetic algorithms come to the later evolution process, individuals of the population might trap into local minima especially for the optimization problems with a big problem space and many minima. Hence, it is more possible that the fitness value related to the current individual is lower than the random search, and it can be expected that two individual neighbors, which are different from each other in the topology, are located in the same concave in the searching space. There are potential genes between the two closest individual neighbors which have lower fitness value than these two individuals.

**Figure 1:**
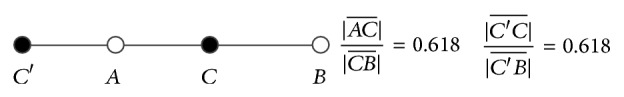
The searching of the opposite golden ratio based local positions.

In recent years, the golden ratio has also been applied to optimize timings of traffic signal systems with good results [16]. Here, the golden ratio is introduced to find the genes of the local search position. As in Figure 1, Point *A* and Point *B* are two adjacent individual neighbors, and Point *C* is the potential local position determined by the golden ratio of *A* and *B*; that is the relationship between the segment *AC* and the segment *AB* satisfies the golden ratio definition. In addition, to expand the local searching area around the current individual positions during the whole searching process, the local position Point *C*′ can also be determined by the opposite golden ratio as the opposition concept has been used in evolution optimization algorithm and good performance was obtained. Here, the concept of the opposite golden ratio is to rotate the Point *C* by 180 degrees, and the relationship between the segment *C*′*C* and the segment *C*′*B* still meets the golden ratio definition.

*(2) Decoding of Decision Variables*. Decision variables of expanded capacity in the upper-level model are considered as an individual. Thus, the chromosome of individuals can be expressed by a vector of decision variables, denoting by $u=(u_{a1},u_{a2},\ldots ,u_{a|\overset{-}{A}|})$. Here, the $\left|\overset{-}{A}\right|$ is the total number of links with expanded capacity.

**Figure 2:**
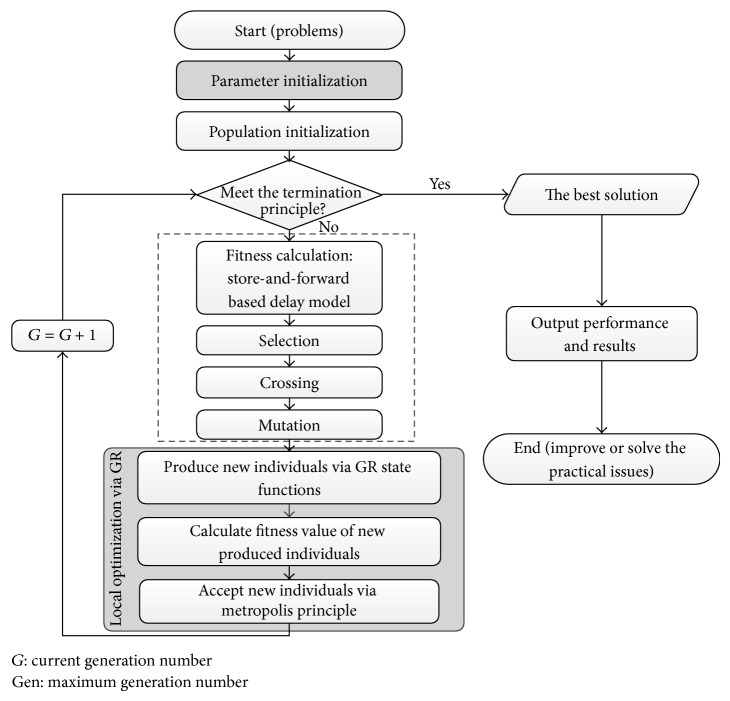
Golden ratio based hybrid genetic algorithm.

*(3) The Hybrid Genetic Algorithm Integrated with Golden Ratio*. The hybrid genetic algorithm integrated with golden ratio (HGAGR) has been developed to obtain optimal solutions for the expanded capacity of available links in the urban road network. The process of the HGAGR is shown in Figure 2.

**Step 1 (initialization).**

Initialize the HGAGR parameters, including population size *M*, evolution generation Gen, GR local optimum size *m*, and the excellent subpopulation size *Ms*. The cross rate and mutation rate are adaptive to the generation and fitness value. Then, generate the real-coded initial population that meets the constraints in formula (5) and the principle of having individuals different from each other.

**Step 2 (fitness evaluation).**

According to the expanded plan of each individual *X*, the OD of a specific network is reassigned by Frank-Wolfe algorithm and then calculates the total network travel time (*Z*) by formula (1), and then the fitness value of this individual is computed in the following formula:

(13) $$\begin{array}{c} f\left(Z\right)=\frac{1}{1+Z}. \end{array}$$

**Step 3 (selection).**

Use the roulette wheel selection.

**Step 4 (crossing).**

Use the nonuniform arithmetic crossover operator.

**Step 5 (mutation).**

Use the nonuniform mutation operator, by which the degree of mutation is still adaptively adjusted with the generation and fitness value. Denote *U*__*nk*__^*i*^ as the *i*th gene on chromosome *k* to be mutated; then descendant *C*__*nk*__^*i*^ is computed in the following formula:

(14) C  nki=U  nki+Δ(t,0−U  nki),if  γ=0,U  nki−Δ(t,Uamax⁡−U  nki),if  γ=1,while,  Δ(t,y)=y·(1−r1/1+n/Gen·(1−f′(Z)/Fmax⁡)b).

In formula (14), *γ* is a 0-1 variable; *f*′(*Z*) is the fitness of individual, *F*_max⁡_ is the maximum fitness in cur-generation, *r* is a random number within [0, 1], and *b* is a genetic parameter to control the degree of dependence on fitness; here *b* is 0.5.

**Step 6 (elitist strategy).**

Replace the worst individual in current-generation with the best one in parent generation.

**Step 7 (golden ratio-based local optimization).**

To increase the population diversity, choose the best *Ms* individuals in the above improved real-coded genetic algorithm to generate an excellent subpopulation, and select the best 2*m* individuals randomly from this subpopulation as initial vector; then conduct the local optimization of the IRGA via the following golden ratio operator.

(1) Denote the excellent pair of individuals as *A* and *B*, respectively, and generate new individuals (*C* and *C*′) by the function of GR as in Figure 1.
(2) Evaluate each new individual by Step 2.
(3) Select these new generated individuals by metropolis principle. The acceptance probability of individual *X*(*P*(*X*)) is calculated by formula (15), in which *P*(*X*) increases parallel with the evolutional generation and fitness value to approximate the better solution in accelerating convergence; that is, the HGAGR gives more belief on local optimization in later evolution process:
  (15) $$\begin{array}{c} P\left(U\right)=e^{-\alpha \left(1-G\cdot f\left(Z\right)/\text{Gen}\right)}. \end{array}$$

In formula (15), *α* is the proportional coefficient that controls the dependent degree on the evolutional generation; here *α* is 0.6.

(4) Replace the current-generation worst individuals of the IRGA by those selected new individuals and then go to Step 8.

**Step 8 (judgment of termination principle).**

If *n* < Gen, go to Step 2. Otherwise, output the best solution and the value of evaluation indices.

## 5. Numerical Analysis

### 5.1. Case Description

**Figure 3:**
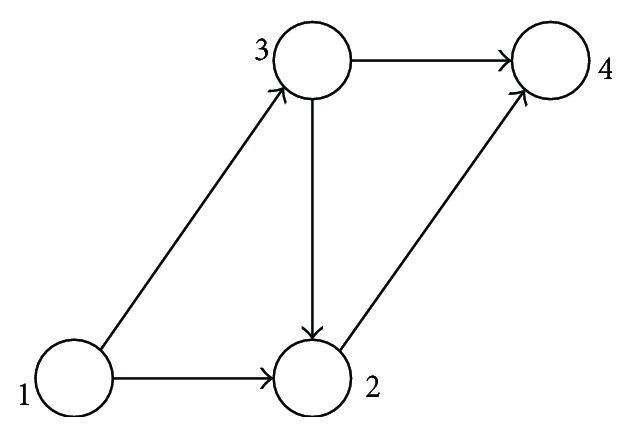
Suwansirikul one-way network.

Use the Suwansirikul one-way network verification to establish the bilevel programming and solve the algorithm [17]. As is shown in Figure 3, the network contains an OD pair. The traffic flow from node 1 to node 4 is 60; *q*_14_ = 60. The values of free flow travel time *t*_0_, section capacity *c*_*a*_, and capacity expansion cost coefficient *τ*_*a*_ are shown in Table 1. The impedance function used is BPR impedance function, whose *α* equals 0.15 and *β* equals 4. *γ* in the objective function is 1.5. The value range of expanded capacity is [0, 30].

The parameter settings of the HGAGR are as follows: population size *M* = 50; generation times Gen = 100; golden section optimal algorithm *m* = 6; subpopulation size *Ms* = 3*m*; dependence of evolution algebra *α* = 0.6; dependence of individual fitness *b* = 0.5.

### 5.2. Result Analysis

*(1) Capacity Expansion of Suwansirikul Road Network*. Flank-Wolfe algorithm is used to divide the specified OD capacity into each link section in the lower-level model. Then HGAGR are used to calculate the expanded capacity in the upper-level model. The statistics result of flow of each section *v*_*a*_, section saturation *v*_*a*_/*c*_*a*_, and network total travel time, under the circumstance of equalization *T*_all_ = ∑_*a*∈*A*_ *t*_*a*_ · *v*_*a*_, are shown in Table 2.

It shows that the capacity expansion is not significant compared to the SN. The reasons are as follows. (1) The traffic demand of former SN is small. In the state of equilibrium, the saturation of each section satisfies the network design level. The saturation of all sections are less than 1. (2) The Lagrangian multiplier *γ* is too large, which leads to the high cost of improving total travel time.

*(2) Influencing Factors Analysis*. With the increase of traffic demand, network capacity needs expanding [18]. To model the real network environment, this paper firstly increases the demand of the SN by *q*_14_ = 120 and gets the new Suwansirikul network with increased demand (SND). Then the expended capacity of the SND is reoptimized with *γ* = 0.03. Calculation results of two scenarios are shown in Table 3. In the SND, the network capacity of each section has increased to meet the need of travel demand, but the sections are still in a state of saturation. As the difference of *γ* and the cost of capacity expansion is too large, the cost of traffic congestion is higher than the cost of increasing the capacity when the system is optimal. As *γ* = 0.03, the cost of capacity expansion may be reduced, and the saturation of each section will improve. With the limited budget and increased travel demand, the traffic condition can be improved by expanding the network capacity. Supposing the capacity of each road is 20 pcu, on the basis of the expanding capacity of sections 42 and 43, one road should be added between section 42 and 43, in order to keep the whole network in high service level.

**Figure 4:**
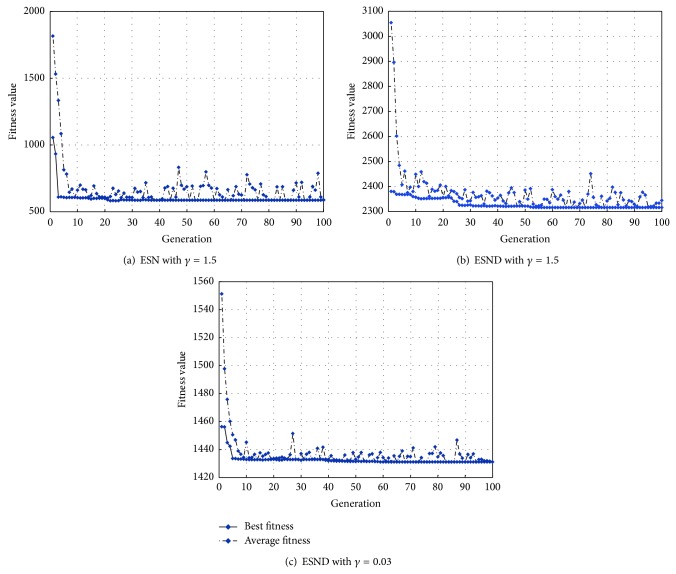
Convergence curves of the HGAGR.

*(3) HGAGR Algorithm Performance*. The convergence curves of the HGAGR under three conditions are shown in Figure 4. The HGAGR converges quickly in first 10 iterations. The objective function value significantly reduces. Then the convergence speed is getting lower and there are few fluctuations of the best fitness values after 50 iterations. During the process of the optimization, the average fitness of the population is fluctuating because of the golden ratio based local search of the HGAGR. The average computation time of the solution algorithm is 122 s, which satisfies the real-time demand of road network evaluation.

## 6. Conclusion

Network capacity represents the level of road network construction and reflects the level of service of the existing road infrastructures. Thus, it is an important decision variable to determine the saturation level, potential capacity, and bottlenecks of existing road network. Aiming at crucial issues of capacity expansion of road network, this paper employed the bilevel mathematical programming modelling for the capacity expansion in the continuous network design problem. To improve the local search ability of simple genetic algorithms, a golden ratio based hybrid genetic algorithm was developed to solve the upper-level model of expanded capacity, and the lower-level model was solved by the classic Frank-Wolfe algorithm. Three numerical analyses on Suwansirikul network indicate the following.

- For capacity expansion, urban road saturation (*v*/*c*) is a key parameter to evaluate the level of road service. When the road saturation is over 0.9, these saturated roads become bottleneck sections. To meet increasing traffic demand, reconstruction of road facilities is necessary; that is to say, for bottleneck sections, increasing link capacity with building new lane or new link can balance traffic distribution in whole network with better use of total network capacity.
- For the algorithm performance, the proposed HGAGR is more time-efficient because of less calculations and simpler convergence condition. The good performance of the proposed model also indicates that the HGAGR has the potential in finding reliable solutions with golden ratio based local search around the excellent individuals, instead of random search. Therefore, the design of the improvement measures used to enhance the local search capacity of simple genetic algorithms will be a critical operational issue.

## Figures and Tables

**Table 1:** Section attribution, objective function, and impedance function.

Section	*t*_0_	*c*_*a*_	τ_*a*_
1 → 2	6	30	2.0
1 → 3	4	50	2.0
2 → 4	3	30	2.0
3 → 2	2	20	1.5
3 → 4	5	40	2.0
Objective function	$\underset{u}{\min\;}Z=\sum_{a\in A}t_{a}\left(v_{a},u_{a}\right)\cdot v_{a}+1.5\tau_{a}{\left(u_{a}\right)}^{2},\;a\in A$		
Impedance function	$t_{a}=t_{0}\left[1+\alpha {\left(\frac{v_{a}}{c_{a}*0.9}\right)}^{\beta }\right],\;a\in A$		

**Table 2:** Capacity expansion of Suwansirikul road network (*q*_14_ = 60, *γ* = 1.50).

Section	Network	*t*_*a*_	*v*_*a*_	*c*_*a*_	*v*_*a*_/*c*_*a*_
1 → 2	SN^*^	6.3171	20.8026	30	0.6934
	ESN^*^	6.3147	20.8590	30 + 0.1388	0.6921
1 → 3	SN	4.3454	39.1974	50	0.7839
	ESN	4.3377	39.1410	50 + 0.2118	0.7795
2 → 4	SN	3.5069	27.8157	30	0.9272
	ESN	3.4890	27.8241	30 + 0.2794	0.9189
3 → 2	SN	2.0069	7.0131	20	0.3507
	ESN	2.0067	6.9651	20 + 0.0220	0.3479
3 → 4	SN	5.4791	32.1843	40	0.8046
	ESN	5.4676	32.1759	40 + 0.2333	0.7997
*T*_all_ = ∑_*a*∈*A*_ *t*_*a*_ · *v*_*a*_	SN	589.7041			
	ESN	588.4805			
$\underset{u}{\min\;}Z=T_{\text{all}}+\gamma \sum_{a\in A}g_{a}\left(u_{a}\right)$	SN	—			
	ESN	589.0714			

^*^ SN: Suwansirikul network; ESN: expanded capacity based Suwansirikul network.

**Table 3:** Capacity expansion of Suwansirikul network with increased demand (*q*_14_ = 120).

Section	ESND^*^	*t*_*a*_	*v*_*a*_	*c*_*a*_ + *u*_*a*_	*v*_*a*_/*c*_*a*_
1 → 2	γ = 1.5	9.9929	43.6304	30 + 3.4030	1.3062
	γ = 0.03	6.8201	47.6516	30 + 24.1917	0.8793
1 → 3	γ = 1.5	7.7518	76.3696	50 + 3.6607	1.4232
	γ = 0.03	4.7896	72.3484	50 + 25.0548	0.9639
2 → 4	γ = 1.5	7.6614	56.2795	30 + 4.8563	1.6146
	γ = 0.03	3.7959	59.4167	30 + 27.2468	1.0379
3 → 2	γ = 1.5	2.0730	12.6491	20 + 0.0099	0.6321
	γ = 0.03	2.0446	11.7651	20 + 1.0465	0.5590
3 → 4	γ = 1.5	9.7827	63.7205	40 + 4.5538	1.4302
	γ = 0.03	5.8275	60.5833	40 + 25.6811	0.9224
*T*_all_ = ∑_*a*∈*A*_ *t*_*a*_(*v*_*a*_, *u*_*a*_) · *v*_*a*_	γ = 1.5	2108.8			
	γ = 0.03	1274.1			
$\underset{u}{\min\;}Z=T_{\text{all}}+\gamma \sum_{a\in A}g_{a}\left(u_{a}\right)$	γ = 1.5	2316.7			
	γ = 0.03	1431.1			

^*^ESND: expanded capacity based Suwansirikul network with increased demand.

## Notations

### List of Key Variables Used in the Network Capacity Models

*A*: Set of links in the network
$\overset{-}{A}$: Set of links with the expanded capacity in the network
$\left|\overset{-}{A}\right|$: The total number of links with the expanded capacity
*W*: Set of OD pairs. For each OD pair numbered *w*, *w* ∈ *W*
*R*_*w*_: Set of routes between the OD pair numbered *w*. For each route *r*, *r* ∈ *R*_*w*_
*f*_*r*_^*w*^: Flow volume of route *r* between the OD pair numbered *w*
**f**: Route flow volume vector **f** = (…,*f*_*r*_^*w*^,…)^*T*^ in the lower model
*v*_*a*_: Link flow volume, *a* ∈ *A*
**v**: Link flow volume vector **v** = (…, *v*_*a*_,…)^*T*^ in the lower model
*u*_*a*_: Top decision variables of the expanded capacity of link *a*, *a* ∈ *A*
*u*_*a*_^max⁡^: The upper limit of the expanded capacity of link *a*, *a* ∈ *A*
**u**: The expanded link capacity vector in the top model $\left(u_{a1},u_{a2},\ldots ,u_{a|\overset{-}{A}|}\right)$
*t*_*a*_(*v*_*a*_, *u*_*a*_): Travel time of link *a* ∈ *A*, which is an equation of link flow volume and expanded link capacity
*c*_*r*_^*w*^: Travel time of route *r* between the OD pair numbered *w*
*π*_*w*_: Minimum travel time between the OD pair numbered *w*
*q*_*w*_: Traffic demands between the OD pair numbered *w*
**q**: Vector of demands between all OD pairs (*q*_1_, *q*_2_,…, *q*_*W*_)
*g*_*a*_(*u*_*a*_): Cost of the expanded link capacity, *a* ∈ *A*
*δ*_*ar*_^*w*^: If link *a* is included in route *r* between the OD pair numbered *w*, it equals 1, otherwise it equals 0
*B*: Fixed investment budget of network capacity expansion.

## References

[1] Yang H., Bell M. G. H. (1998). A capacity paradox in network design and how to avoid it. *Transportation Research Part A: Policy and Practice*.

[2] Porta S., Crucitti P., Latora V. (2006). The network analysis of urban streets: a dual approach. *Physica A: Statistical Mechanics and its Applications*.

[3] Lämmer S., Gehlsen B., Helbing D. (2006). Scaling laws in the spatial structure of urban road networks. *Physica A: Statistical Mechanics and Its Applications*.

[4] Masuya Y., Saito K. (1989). Application of T-region in a linear programming problem to the calculation of zonal trip generation and attraction. *Proceedings of the Japan Society of Civil Engineers*.

[5] Wu Y. H., Miller H. J. (2001). Computational tools for measuring space-time accessibility within dynamic flow transportation networks. *Journal of Transportation and Statistics*.

[6] Fisk C. (1980). Some developments in equilibrium traffic assignment. *Transportation Research Part B: Methodological*.

[7] Daganzo C. F., Sheffi Y. (1977). On stochastic models of traffic assignment. *Transportation Science*.

[8] Chen A., Yang C. (2004). Stochastic transportation network design problem with spatial equity constraint. *Journal of the Transportation Research Board*.

[9] Asakura Y., Kashiwadani M. (1993). Estimation model of maximum road network capacity with parking constraints and its application. *Infrastructure Planning Review*.

[10] Yang H., Bell M. G. H., Meng Q. (2000). Modeling the capacity and level of service of urban transportation networks. *Transportation Research Part B: Methodological*.

[11] Yang H., Bell M. G. H. (1998). Models and algorithms for road network design: a review and some new developments. *Transport Reviews*.

[12] Yin Y. (2000). Genetic-algorithms-based approach for bilevel programming models. *Journal of Transportation Engineering*.

[13] Fukushima M. (1984). A modified Frank-Wolfe algorithm for solving the traffic assignment problem. *Transportation Research—Part B Methodological*.

[14] Yang W. C., Zhang L., He Z. C., Zhuang L. J. Optimized two-stage fuzzy control for urban traffic signals at isolated intersection and Paramics simulation.

[15] Yang W., Zhang L., Shi H. A golden ratio-based genetic algorithm and its application in traffic signal timing optimization for urban signalized intersections.

[16] Suwansirikul C., Friesz T. L., Tobin R. L. (1987). Equilibrium decomposed optimization: a heuristic for the continuous equilibrium network design problem. *Transportation Science*.

[17] Meng Q., Yang H. (2002). Benefit distribution and equity in road network design. *Transportation Research Part B: Methodological*.

[18] LeBlanc L. J. (1988). Transit system network design. *Transportation Research Part B: Methodological*.

